# Multi-directional nature of falls among older adults: A rationale for prevention and management

**DOI:** 10.3389/fpubh.2023.1117863

**Published:** 2023-02-21

**Authors:** Matthew Lee Smith, Marcia G. Ory

**Affiliations:** School of Public Health, Texas A&M University, College Station, TX, United States

**Keywords:** falls, fall prevention and management, older adults, intervention, evidence-based practice, multi-level intervention

## 1. Falls as a focal issue for older adults

The global aging population is larger than ever before (1), and it is estimated that 155 countries will have an aging society by the year 2050 (2). In the United States alone, there are more than 50 million adults ages 65 years and older, with this sub-population projected to exceed more than 80 million by 2040 (3). The growing aging population reflects longer life expectancies largely attributed to a combination of medical advancements, accessible healthcare, and supportive and inclusive physical and social environments (4). While there is much variability in the aging process (5), the expansive older adult population brings with it increased prevalence rates of chronic conditions and other health issues (e.g., injurious falls, cognitive decline, malnutrition, mental illness, and social disconnectedness) that will further strain the already over-burdened healthcare system. While there are many pressing and costly geriatric conditions deserving of increased and immediate attention, we will focus on older adult falls as an example of a globally-recognized, age-related condition with a host of negative, but potentially preventable, sequelae (6, 7). Too often falls are narrowly viewed as a natural and inevitable part of aging, which cannot be prevented or managed. The complex and multi-factorial circumstances resulting in a fall require a more holistic view of the event (i.e., causes, facilitators, and contributors) and the older adults' physical, mental, environmental, and medical context. We contend that falls is among the most germane health issues facing older adults because a fall can represent a constellation of interwoven health events and may be centric to multi-level solutions spanning research, healthcare practice, community programming, and policy. This article aims to expand the lens through which we view falls as a public health issue by: (a) offering insights about the upstream indicators and downstream ramifications associated with falls; and (b) highlighting opportunities for interdisciplinary and cross-sectorial solutions to predict, prevent, and manage falls among older adults.

Falls in the United States are the leading cause of unintentional injuries among older adults age 65 years and older (8). The incidence of falls is substantial, with one-in-four older adults falling each year (9). Older adults who fall have an increased risk for injury, reduced physical function, loss of independence, institutionalization, and death (9, 10). The estimated annual direct medical costs of falls exceed $50 billion USD (11), which does not account for the associated costs for rehabilitation, caregiving, skilled nursing, or other resulting health issues (physical or mental). The ramifications of a fall dramatically impact the older adult, but when considered collectively, these negative consequences impose extreme duress on their families, social structures, and the clinical and community-based organizations that serve them. Although the likelihood of falling increases with age, it is not a natural part of aging. Falls are not inevitable; rather, they are largely preventable. The causes of falls are vast, individualistic, situational, and often interrelated. They include intrinsic and extrinsic factors, which are biological, behavioral, and environmental (12).

**Figure 1:**
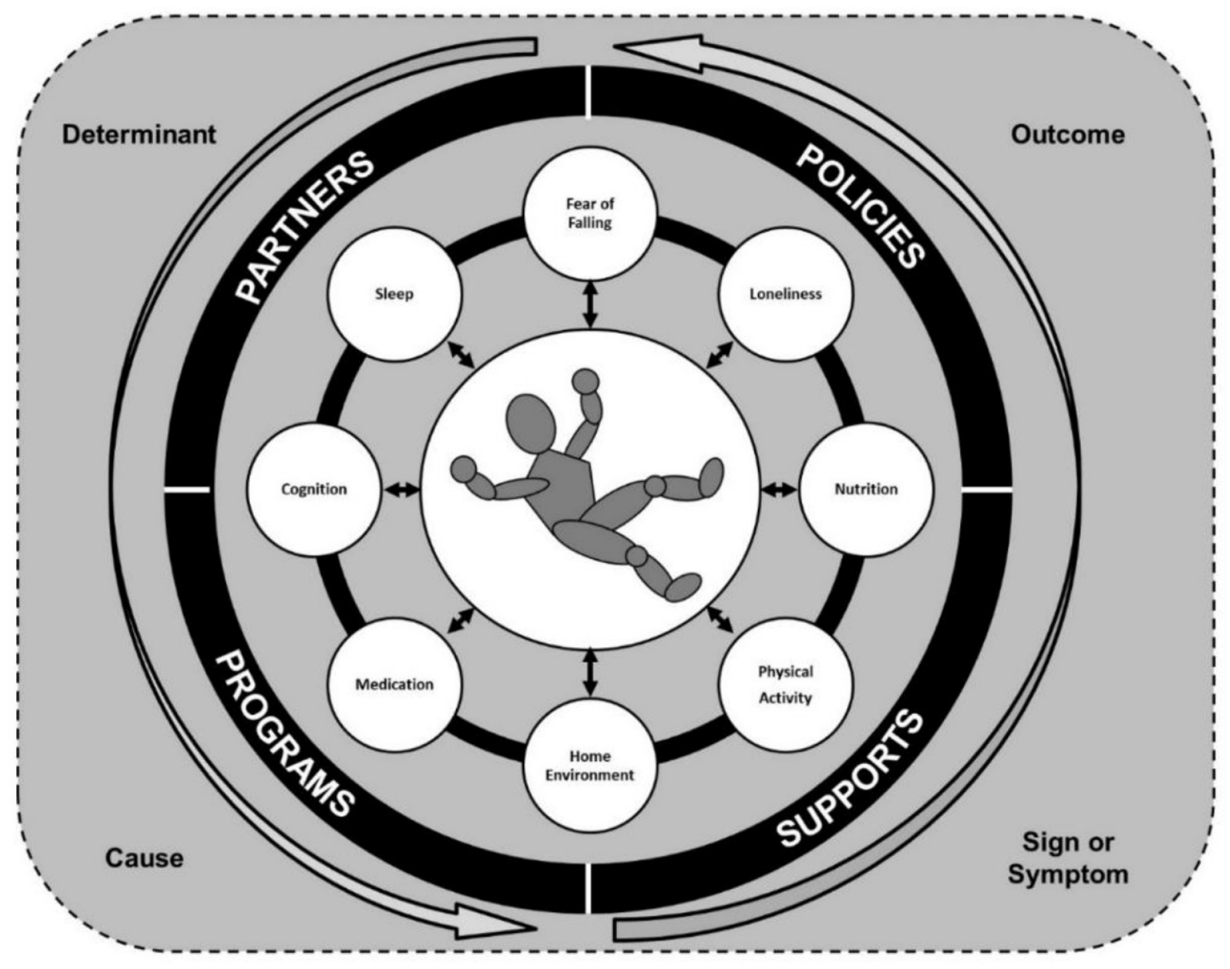
Falls as a multi-directional risk among older adults.

Given the common, complex, and severe nature of falls among older adults, we need to challenge the way we think about falls (13, 14). As researchers, clinicians, and community service providers, we should view falls as a multi-directional occurrence and recognize that the fall itself may not be the focal issue. As depicted in Figure 1, falls can be expressed in multiple ways and take on many roles within the lives of older adults. A fall can be an outcome that is the result of a set of personal or environmental circumstances, a sign or symptom of an underlying health issue, and/or a cause of or trigger for subsequent health consequences. As such, when an older adult presents with a fall, we must holistically assess the person and event to determine the role of the fall within the situational context. There is a need to screen the older adult, perform physical and cognitive assessments, and ask questions of the family and loved ones to understand the situational context and address the upstream and downstream correlates of the fall.

## 2. Select examples of multi-directional fall-related risk

In many instances, a contributing reason for a fall (risk factor) is also affected after a fall. This creates a scenario where the same risk factor can contribute to a fall, emerge after a fall occurs, or intensify after a fall when present prior to a fall. As such, there are opportunities to address these factors both before and after a fall, yet the strategies and interventions may be consistent or uniquely tailored based on the context of fall and other existing risks/complications. To illustrate this point, we provide six selective examples below to describe the multi-directionality of these risk factors as related to fall prevention (before) and management (after). However, it must be recognized that after a fall occurs, both management and prevention efforts are needed because part of managing a fall is preventing a repeated or subsequent fall.

*Fear of falling* is a known risk factor for falling because the fear itself causes older adults to avoid performing certain actions, movements, and activities (15). This fear, in turn, often results in muscular atrophy and reduced lower limb strength/balance/flexibility, which can contribute to the older adult being unsteady on their feet when they are mobile and attempt to perform certain activities. While fear of falling can contribute to a fall, the fear of falling can emerge or escalate after an older adult experiences a fall (16), especially in instances where an injury occurred. Many fall prevention interventions now include attention to strategies to acknowledge and reduce fear of falling.

*Physical activity* is a protective factor against falls in that it promotes circulatory health and lower extremity strength, balance, and flexibility (17). A variety of physical activities and exercises are recommended to promote postural stability and fall prevention, which should be performed consistently over time and become progressively more challenging for the older adult over time (depending on their functional mobility and ability levels). While physical activity is largely protective, in some instances, it can introduce opportunities for falling among older adults based on internal, situational, and environmental factors. After a fall, there is an increased likelihood of physical inactivity or sedentary behavior, especially if the fall resulted in a fracture (e.g., hip, pelvis, and femur) or traumatic brain injury. As such, it is essential to promote physical activity at all ages but allow an older adult ample time to rest and recuperate after a fall before introducing rehabilitation activities (i.e., to regain physical mobility and avoid lower extremity atrophy).

*Medication, and especially polypharmacy*, is a risk factor for falling because the side effects may influence blood pressure and other biological functions that can cause dizziness and postural hypotension (18). When an older adult takes multiple medications simultaneously, the opportunities for negative interaction effects amplify, as can the associated side effects, that can cause decreased consciousness (e.g., blackouts and fainting) or general postural unsteadiness. After a fall occurs, especially a fall with injury, the activities and lifestyle behaviors of a person may change during recovery. They may be prescribed prescription pain relievers, which may create interaction effect issues with existing medication regimens and medication non-adherence issues (e.g., dose, timing, and food accompaniment). Further, the biological processing of the medication may change after a fall because of resulting changes in mobility, diet (appetite suppression from prescription pain relievers), and sleep patterns. Increasing recognition of these medication-related risks have prompted doctors and pharmacists to perform routine checks of medication usage, and cross-reference for potential interaction effects, during each medical encounter.

*Nutrition, and especially malnutrition*, is an important and often unrecognized risk factor for falls and lower limb functioning (19–22). Protein-rich diets among older adults, which impact muscle mass and strength, may prevent the occurrence of a fall as well as accelerate the recovery time after an injurious fall (23, 24). Proper and balanced diets can provide the bones and muscles sufficient strength and functioning needed to accommodate to changing functional capacities with aging. Beyond access to healthful and affordable foods, older adults may benefit from nutritional supplements to bolster daily protein consumption and muscular function.

*Loneliness, and overall social disconnectedness*, is a risk factor for falls because older adults feeling isolated from others may be socially excluded or have limited support networks, thus hindering their social activities and exacerbating other risk factors associated with falls (e.g., depression, cognitive impairment, sedentary behavior, and substance use) (25). After a fall, older adults may become homebound or have limited community-based mobility, which can hinder their ability to perform instrumental activities of daily living and limit meaningful interactions with others, which can cause feelings of loneliness, isolation, and disconnectedness. This situation can be especially impact older adults without robust social networks to meet their needs and keep them socially engaged (e.g., those residing alone).

*Home and Community Environments* are seen as a major extrinsic risk factors for falls both within the home and in the community (26, 27). Environmental factors can be associated with common trips and slips among older adults with declining physical capacities to compensate for environmental hazards, while environmental modifications can reduce fall-related risk by introducing handrails, grab bars, and ample lighting within homes and areas frequented by older adults (28). Similarly, to reduce falls risk, there is greater appreciation of the importance of having safe, navigable outdoor environments with amenities such as crosswalks/pedestrian signals, seating for rest, and lighting/shade in recreational areas and places where older adults engage in utilitarian activities (29, 30). After a fall, older adults may be unable to drive or travel freely within their community (e.g., due to injury, immobility, or pain medication), which may limit their ability to run errands or go to doctor's appointments necessary for rehabilitation. Further, they may be unable to clean or pick-up clutter around the home, which may create or contribute to in-home fall-related hazards.

## 3. Solutions to advance fall prevention and management

Like many geriatric health issues, fall-related risks are multi-factorial and therefore require multi-factorial solutions and a diverse, multi-disciplinary team/workforce to: (a) reduce incidence and prevalence rates; and (b) help older adults overcome the fall after it occurs. While the fall itself is a focal point for intervention, it is often indicative of other issues occurring within the older adult's life, which highlights our need to recognize and address the complexities of upstream indicators and downstream ramifications. Therefore, we must be simultaneously reactive and proactive to address falls and fall-related risk comprehensively within our communities.

Multi-level approaches are needed to adequately and comprehensively prevent and manage falls among older adults. At the individual level, efforts are needed to routinely assess fall-related risk among older adults independently from, and within the context of, other known risk factors. As an example, in the United States, the Centers for Disease Control and Prevention (CDC) developed a comprehensive toolkit, STopping Elderly Accidents, Deaths, and Injuries (STEADI) to help healthcare professionals screen and assess for fall-related risk and make tailored referrals to specialists and community-based programs (31). While fall screening is useful in the falls context, additional efforts are needed to uniformly assess other associated risk factors such as malnutrition, sedentary behavior, loneliness, physical and cognitive functioning, and sleep problems. Such risk inventories interpreted in a falls context, and reviewed with multi-disciplinary teams in healthcare and community settings can elevate fall prevention efforts and assist to mitigate fall-related ramifications, if a fall occurs. The amassing body of intervention research confirms that the most successful fall prevention programs are those that are multifactorial and multicomponent (32). As such, within the United States' aging services network, there are many evidence-based fall prevention programs disseminated at the national, state, and local levels (33). These interventions are critical to address intrinsic and extrinsic fall-related risks to prevent and manage older adult falls. However, to reach a sufficient number of older adults with effective programming, a diverse delivery infrastructure is needed that includes healthcare, community non-profit, residential, faith-based, government, education, and industry partners (34, 35). Then, after establishing infrastructure, dedicated efforts and funding are needed for program scalability and sustainability (36). At the community level, the creation of national- and state-based coalitions (or task forces, action alliances, etc.) to address falls and other issues facing older adults have great potential to unify efforts and embed evidence-based solutions and best practices across communities over time and geography (37–39). Further, the United States has an active age-friendly communities movement, which encourages attention to environmental and policy issues to support livable and connected communities that facilitate increased physical activity, access to resources, and social interactions (i.e., thereby ameliorating important fall risks) (40, 41).

The Otago Exercise Program (OEP) is an example of an effective fall prevention program that fits within a multi-level fall prevention framework and fosters multi-sectorial collaboration and action (13, 42). Adopted in the United States from New Zealand (43, 44), OEP is an innovative model of in-home, one-on-one physical therapy sessions targeting frailer older adults. These home-bound older adults are the primary audience for this program because they are typically older, have more comorbidities, face more environmental and social challenges, and are at greater risk for recurring falls. Based on the older adult's risk and ability levels, the physical therapist engages participants in 17 strength and balance exercises over a series of six visits. The intensity of exercises is progressively increased to improve older adults' lower-limb strength, balance, and flexibility. OEP is complemented by a weekly walking program to assist participants be more active between physical therapy visits and encourage mobility outside the home (45). Older adults are often referred to OEP by healthcare providers following a clinical risk assessment (e.g., STEADI) or being discharged after a fall (46). After participating in OEP, participants are often referred to other evidence-based fall prevention programs in community settings (e.g., A Matter of Balance, Stepping On, Tai Chi: Moving for Better Balance) so they can continue to mitigate fall-related risk and become more physically active. OEP has been shown to significantly improve participants' functional performance (i.e., Timed Up-and-Go, 30-Second Chair Stand, Four-Stage Balance Test) (47, 48). Based on these outcomes, OEP was translated so it could reach more older adults and be implemented in more clinical and community settings. As a result, OEP has been translated for delivery in group and community settings, facilitation by non-physical therapists (e.g., certified occupational therapy assistants, lay leaders of evidence-based programs) (48, 49), and virtual dissemination using an avatar-guided platform (50). These translated OEP versions have also demonstrated effectiveness, respectively. The delivery and evolution of OEP illustrate the importance of cross-sectoral screening, referral, and intervention to identify frail home-bound older adults needing assistance and assist them prevent and overcome falls.

Offering multi-level, multi-sectoral solutions in the United States is consistent with the global initiatives promulgated by the United Nations' Decade of Healthy Aging (2021–2030), which gives concrete actions for making healthy aging a global reality (51). Because falls are a central and complex health issue impacting the older population, coordinated multi-level research, practice, and policy efforts are needed to understand the many roles of falls, its drivers, and its consequences. Considering a fall can be an outcome, sign or symptom, or cause or trigger, it is necessary that we examine the context of each fall among older adults to gain a holistic view of their health risks and conditions. As a society, at all levels, we must proactively screen, assess, refer, intervene, treat, and follow-up with our older adults to prevent falls and protect older adults from related (and potentially preventable) sequelae. In the coming decade, we must view and prioritize falls as a pressing health issue within the aging population and collectively advocate for multi-level solutions spanning research, healthcare practice, community programming, and policy.

## 4. Conclusions

The situations and circumstances surrounding a fall are complex and should be viewed as a multi-directional occurrence. Researchers, practitioners, and policy makers must challenge the way they think about falls and recognize that the fall itself may not be the focal issue. Collectively, we must assess the upstream indicators and downstream ramifications of falls and identify practical and effective solutions to address these factors both before and after a fall. While we have implemented strategies to identify falls in clinical settings and refer at-risk older adults to evidence-based fall prevention programs across community sectors, we must also consider how falls fit within the greater context of other health issues (e.g., malnutrition, physical inactivity, cognitive impairment, social disconnectedness, polypharmacy, and the built environment). Despite our many advancements, additional multi-level, multi-sectorial solutions are needed to prevent and manage falls among the growing and diverse global aging population. Our efforts must span research, healthcare practice, community programming, and policy to ensure solutions are practical, effective, replicable, scalable, and sustainable.

## Author contributions

MS conceptualized and wrote the article. MO wrote the article. All authors reviewed and approved the submitted manuscript.

## References

[1] World Health Organization. Ageing and Health. (2022). Available online at: https://www.who.int/news-room/fact-sheets/detail/ageing-and-health#:~:text=At%20this%20time%20the%20share,2050%20to%20reach%20426%20million (accessed November 1, 2022).

[2] United Nations Department Department of Economic Social Affairs Population Division. (2019). World Population Ageing 2019 - Highlights. Available online at: https://www.un.org/en/development/desa/population/publications/pdf/ageing/WorldPopulationAgeing2019-Highlights.pdf (accessed November 1, 2022).

[3] Administration for Community Living. Projected Future Growth of Older Population. (2022). Available online at: https://acl.gov/aging-and-disability-in-america/data-and-research/projected-future-growth-older-population (accessed November 1, 2022).

[4] McMaughan DJ Oloruntoba O Smith ML. Socioeconomic status and access to healthcare: Interrelated drivers for healthy aging. Front Public Health. (2020) 8:231. 10.3389/fpubh.2020.00231 32626678 PMC7314918

[5] Ory MG Smith ML. What if healthy aging is the “new normal”? Int J Environ Res Public Health. (2017) 14:1389. 10.3390/ijerph14111389 29140264 PMC5708028

[6] Mackenzie L Tan MP Silveria Gomes AR Dennis S Pit SW Wales Smith ML. Falls Prevention for Older People in Primary Care Settings. (2022). Available online at: https://www.frontiersin.org/research-topics/19002/falls-prevention-for-older-people-in-primary-care-settings (accessed November 1, 2022).

[7] Frieson CW Tan MP Ory MG Smith ML. Evidence-based practices to reduce falls and fall-related injuries among older adults. Front Public Health. (2018) 6:222. 10.3389/fpubh.2018.00222 30186826 PMC6110876

[8] Centers for Disease Control Prevention. Injury Prevention and Control. Keep on Your Feet – Preventing Older Adult Falls. (2020). Available online at: https://www.cdc.gov/injury/features/older-adult-falls/index.html (accessed November 1, 2022).

[9] Centers for Disease Control Prevention. Older Adult Fall Prevention. Facts about Falls. (2021). Available online at: https://www.cdc.gov/falls/facts.html (accessed November 1, 2022).

[10] Vaishya R Vaish A. Falls in older adults are serious. Indian J Orthop. (2020) 54:69–74. 10.1007/s43465-019-00037-x 32257019 PMC7093636

[11] Centers for Disease Control Prevention. Older Adult Fall Prevention. Cost of Older Adult Falls. (2020). Available online at: https://www.cdc.gov/falls/data/fall-cost.html (accessed November 1, 2022).

[12] Centers for Disease Control Prevention. Fact Sheet – Risk Factors for Falls. (2017). Available online at: https://www.cdc.gov/steadi/pdf/Risk_Factors_for_Falls-print.pdf (accessed November 1, 2022).

[13] Shubert TE Smith ML Schneider EC Wilson AD Ory MG. Commentary: Public health system perspective on implementation of evidence-based fall-prevention strategies for older adults. Front Public Health. (2016) 4:252. 10.3389/fpubh.2016.00252 27900316 PMC5111173

[14] Shubert TE Smith ML Prizer LP Ory MG. Complexities of fall prevention in clinical settings: A commentary. Gerontologist. (2014) 54:550–8. 10.1093/geront/gnt079 23887933

[15] Schoene D Heller C Aung YN Sieber CC Kemmler W Freiberger E. a systematic review on the influence of fear of falling on quality of life in older people: Is there a role for falls? Clin Interv Aging. (2019) 14:701. 10.2147/CIA.S197857 31190764 PMC6514257

[16] Lavedán A Viladrosa M Jürschik P Botigué T Nuín C Masot O. Fear of falling in community-dwelling older adults: A cause of falls, a consequence, or both? PLoS ONE. (2018) 13:e0194967. 10.1371/journal.pone.0194967 29596521 PMC5875785

[17] van Gameren M Hoogendijk EO van Schoor NM Bossen D Visser B Bosmans JE. Physical activity as a risk or protective factor for falls and fall-related fractures in non-frail and frail older adults: A longitudinal study. BMC Geriatr. (2022) 22:1. 10.1186/s12877-022-03383-y 35996101 PMC9396867

[18] Ie K Chou E Boyce RD Albert SM. Fall risk-increasing drugs, polypharmacy, and falls among low-income community-dwelling older adults. Innov Aging. (2021) 5:igab001. 10.1093/geroni/igab001 33644415 PMC7899132

[19] Eckert CD Tarleton EK Pellerin J Mooney N Gell NM. Nutrition risk is associated with falls risk in an observational study of community-dwelling, rural, older adults. J Aging Health. (2022) 29:8982643221096944. 10.1177/08982643221096944 35487237 PMC10370346

[20] Corish CA Bardon LA. Malnutrition in older adults: Screening and determinants. Proc Nutr Soc. (2019) 78:372–9. 10.1017/S0029665118002628 30501651

[21] Esquivel MK. Nutritional assessment and intervention to prevent and treat malnutrition for fall risk reduction in elderly populations. Am J Lifestyle Med. (2018) 12:107–12. 10.1177/1559827617742847 30283246 PMC6124993

[22] Lackoff AS Hickling D Collins PF Stevenson KJ Nowicki TA Bell JJ. The association of malnutrition with falls and harm from falls in hospital inpatients: Findings from a 5-year observational study. J Clin Nurs. (2020) 29:429–36. 10.1111/jocn.15098 31715045

[23] Coelho-Júnior HJ Milano-Teixeira L Rodrigues B Bacurau R Marzetti E Uchida M. Relative protein intake and physical function in older adults: A systematic review and meta-analysis of observational studies. Nutrients. (2018) 10:1330. 10.3390/nu10091330 30235845 PMC6163569

[24] Ten Haaf DS Nuijten MA Maessen MF Horstman AM Eijsvogels TM Hopman MT. Effects of protein supplementation on lean body mass, muscle strength, and physical performance in nonfrail community-dwelling older adults: A systematic review and meta-analysis. Am J Clin Nutr. (2018) 108:1043–59. 10.1093/ajcn/nqy192 30475963

[25] Petersen N König HH Hajek A. The link between falls, social isolation and loneliness: A systematic review. Arch Gerontol Geriatr. (2020) 88:104020. 10.1016/j.archger.2020.104020 32018091

[26] Clemson L Stark S Pighills AC Torgerson DJ Sherrington C Lamb SE. Environmental interventions for preventing falls in older people living in the community. Cochr Datab Systemat Rev. (2019) 2019:CD013258. 10.1002/14651858.CD013258 PMC9998238 36893804

[27] Lee S. Falls associated with indoor and outdoor environmental hazards among community-dwelling older adults between men and women. BMC Geriatr. (2021) 21:1–2. 10.1186/s12877-021-02499-x 34641812 PMC8507100

[28] Centers for Disease Control Prevention. Check for Safety: A Home Prevention Checklist for Older Adults. (2015). Available online at: https://www.cdc.gov/steadi/pdf/check_for_safety_brochure-a.pdf (accessed November 1, 2022).

[29] Lee C Lee C Stewart OT Carlos HA Adachi-Mejia A Berke EM. Neighborhood environments and utilitarian walking among older vs. younger rural adults. Front Public Health. (2021) 2021:532. 10.3389/fpubh.2021.634751 34150697 PMC8211879

[30] Li W Procter-Gray E Lipsitz LA Leveille SG Hackman H Biondolillo M. Utilitarian walking, neighborhood environment, and risk of outdoor falls among older adults. Am J Public Health. (2014) 104:e30–7. 10.2105/AJPH.2014.302104 25033118 PMC4151909

[31] Centers for Disease Control Prevention. STEADI – Older Adult Fall Prevention. (2021). Available online at: https://www.cdc.gov/steadi/index.html (accessed November 1, 2022).

[32] Hopewell S Adedire O Copsey BJ Boniface GJ Sherrington C Clemson L. Multifactorial and multiple component interventions for preventing falls in older people living in the community. Cochr Datab Systemat Rev. (2018). 10.1002/14651858.CD012221.pub2 30035305 PMC6513234

[33] Smith ML Towne SD Jr Herrera-Venson A Cameron K Horel SA Ory MG. Delivery of fall prevention interventions for at-risk older adults in rural areas: Findings from a national dissemination. Int J Environ Res Public Health. (2018) 15:2798. 10.3390/ijerph15122798 30544658 PMC6313583

[34] Smith ML Ory MG Ahn S Belza B Mingo CA Towne SD Jr. Reaching diverse participants utilizing a diverse delivery infrastructure: A replication study. Front Public Health. (2015) 3:77. 10.3389/fpubh.2015.00077 25964949 PMC4410486

[35] Towne Jr SD Smith ML Ahn S Altpeter M Belza B Kulinski KP. National dissemination of multiple evidence-based disease prevention programs: Reach to vulnerable older adults. Front Public Health. (2015) 2:156. 10.3389/fpubh.2014.00156 25964901 PMC4410420

[36] Smith ML Durrett NK Schneider EC Byers IN Shubert TE Wilson AD. Examination of sustainability indicators for fall prevention strategies in three states. Eval Program Plann. (2018) 68:194–201. 10.1016/j.evalprogplan.2018.02.001 29621686

[37] Ory MG Towne Jr SD Howell D Quinn C Eblen KJ Swierc SM. Commentary: Working toward a multi-program strategy in fall prevention. Front Public Health. (2017) 5:14. 10.3389/fpubh.2017.00014 28243585 PMC5304425

[38] Schneider EC Smith ML Ory MG Altpeter M Beattie L Scheirer MA. State fall prevention coalitions as systems change agents: An emphasis on policy. Health Promot Practice. (2016) 17:244–53. 10.1177/1524839915610317 26500227

[39] Smith ML Chaudhary S Nieb S Bayakly R Graham K Head E. Commentary: Building the older adult fall prevention movement–Steps and lessons learned. Front Public Health. (2016) 4:277. 10.3389/fpubh.2016.00277 28066756 PMC5177609

[40] Lee C Zhu X Lane AP Portegijs E. Healthy aging and the community environment. Front Public Health. (2021) 9:737955. 10.3389/fpubh.2021.737955 34722446 PMC8553941

[41] AARP. AARP Network of Age-Friendly States and Communities. (2022). AARP Livable Communities. Available online at: https://www.aarp.org/livable-communities/network-age-friendly-communities/ (accessed November 1, 2022).

[42] Smith ML Schneider EC Byers IN Shubert TE Wilson AD Towne Jr SD. Reported systems changes and sustainability perceptions of three state departments of health implementing multi-faceted evidence-based fall prevention efforts. Front Public Health. (2017) 5:120. 10.3389/fpubh.2017.00120 28642861 PMC5462909

[43] Campbell AJ Robertson MC Gardner MM Norton RN Tilyard MW Buchner DM. Randomised controlled trial of a general practice programme of home based exercise to prevent falls in elderly women. Br Med J. (1997) 315:1065–9. 10.1136/bmj.315.7115.1065 9366737 PMC2127698

[44] Campbell AJ Robertson MC Gardner MM Norton RN Buchner DM. Falls prevention over 2 years: A randomized controlled trial in women 80 years and older. Age Ageing. (1999) 28:513–8. 10.1093/ageing/28.6.513 10604501

[45] Shubert TE Smith ML Ory MG Clarke CB Bomberger SA Roberts E. Translation of the Otago Exercise Program for adoption and implementation in the United States. Front Public Health. (2015) 2:152. 10.3389/fpubh.2014.00152 25964899 PMC4410425

[46] Stevens JA Smith ML Parker EM Jiang L Floyd FD. Implementing a clinically based fall prevention program. Am J Lifestyle Med. (2017) 5:1559827617716085. 10.1177/1559827617716085 31903086 PMC6933561

[47] Shubert TE Smith ML Jiang L Ory MG. Disseminating the Otago Exercise Program in the United States: Perceived and actual physical performance improvements from participants. J Appl Gerontol. (2018) 37:79–98. 10.1177/0733464816675422 27794055

[48] Shubert TE Smith ML Goto L Jiang L Ory MG. Otago exercise program in the United States: Comparison of 2 implementation models. Phys Ther. (2017) 97:187–97. 10.2522/ptj.20160236 28204770

[49] Shubert TE Goto LS Smith ML Jiang L Rudman H Ory MG. The otago exercise program: Innovative delivery models to maximize sustained outcomes for high risk, homebound older adults. Front Public Health. (2017) 5:54. 10.3389/fpubh.2017.00054 28386536 PMC5362608

[50] Shubert TE Chokshi A Mendes VM Grier S Buchanan H Basnett J. Stand Tall—A virtual translation of the Otago Exercise Program. J Geriatr Phys Ther. (2020) 43:120–7. 10.1519/JPT.0000000000000203 29958232

[51] World Health Organization. UN Decade of Healthy Ageing. (2022). Available online at: https://www.who.int/initiatives/decade-of-healthy-ageing (accessed November 1, 2022).

